# Assessment of ecosystem services provided by livestock agroecosystems in the tropics: a case study of tropical island environment of Guadeloupe

**DOI:** 10.1007/s11250-021-02880-3

**Published:** 2021-08-15

**Authors:** J.-L. Gourdine, A. Fourcot, C. Lefloch, M. Naves, G. Alexandre

**Affiliations:** 1 Unité de Recherches Zootechniques, INRAE, URZ, 97170 Petit-Bourg, France; 2 Université de Lorraine, INRAE, USC 340, UR AFPA, 54518 Vandœuvre-lès-Nancy, France

**Keywords:** Farm, Typology, Ecosystem services, Local breeds

## Abstract

The present study aims to assess (1) the ecosystem services (ES) provided by LFS and (2) the differential ES between local (Creole) and exotic breeds from pig, cattle and goat. The ES are defined as the benefits that humans derive from LFS. They were summarized in 12 ES indicators that cover services related to provisioning, ecological and socio-cultural aspects and territorial vitality. A total of 106 LFS units that covers the five agroecological zones of Guadeloupe were analysed. Functional typologies of LFS per species were created from surveys. The effect of breed on the ES indicators was tested. Results showed that the 40 pig LFS units were separated into 3 clusters that were differentiated in ES according to provisioning ES (cluster 1), cultural use and sale to the neighborhood (cluster 2) and pork self-consumption (cluster 3). The typology of the 57 farms with cattle distinguished 4 clusters with differences in ES provided in self-consumption (cluster1), ecological ES (cluster 2), socio-cultural ES for racing or draught oxen (cluster 3) and ES associated with territory vitality (cluster 4). The 66 goat LFS units were classified into 3 clusters different in ES concerning self-consumption (cluster 1), cultural aspects (cluster 2) and provisioning ES (cluster 3). Our study highlights that ES indicators are not breed dependent (*P* > 0.10) but rather livestock farming system dependent. The ES rely more on the rearing management than on the breed type, and up to now, there are no specifications in Guadeloupe to differentiate management between breeds.

## Introduction

Livestock farming systems (LFS) are at the centre of the debate on their ability to meet global challenges of food security, the limitation of greenhouse gases and fossil energy consumption and finally the preservation of biodiversity (Poore and Nemecek 2018). Meanwhile, LFS are key players in the development of many rural territories (Dedieu et al. 2011). Indeed, LFS could determine the use of agricultural areas and landscapes, and affect human culture and the economy (Duteurtre and Faye 2009). In this context and in particular, in the tropical conditions of Guadeloupe, there is a need to better understand and characterize the full range of ecosystem services (ES) provided by LFS, defined here as the direct and indirect contributions of LFS to human well-being (TEEB 2010). Based on the methodological framework proposed by Ryschawy et al. (2015), the ES can be classified into bundles of ES including provisioning services (i.e. products), ecological services (e.g. pasture-based systems, feed self-sufficiency), socio-cultural services (e.g. recreation) and territorial vitality (i.e. the rural employment sector). In the tropics, it is often stated that LFS are multifunctional, and the objectives and ES provided by LFS are varied and are not only related to productivity (Alexandre et al. 2014; Hernandez-Castellano et al. 2019). Furthermore, tropical LFS often rely upon local breeds (Hoffmann 2013; FAO 2015). These breeds are well adapted to their environment and their production systems may increase or decrese the values of ES (Nozieres-Petit and Lauvie 2018). The implementation of typologies is a well-known method to describe and understand the heterogeneity between farmers’ practices (Blazy et al. 2009; Alvarez et al. 2018). However, few studies in LFS typologies deal with ES although different types of methods are available to identify and evaluate ES provided by LFS (as reviewed by Bernués and Martin-Collado 2019). In this study, methods combining socio-cultural, biophysical and economic aspects (Ryschawy et al. 2015) were implemented into a typological analysis to identify ES provided by LFS located in the French Caribbean archipelago of Guadeloupe (1434 km^2^, N 16° and W 62°). Another objective of this study was to investigate if the rearing of local breeds modifies the ES provided by LFS.

## Materials and methods

### Study locations

The area covered by the study is the Guadeloupe archipelago. Guadeloupe has a wide range of rainfall, particularly on its two largest islands, Basse-Terre (848 km^2^) and Grande-Terre (586 km^2^). The archipelago of Guadeloupe is characterized by five agroecological regions (AER) (Sierra et al. 2015). In the volcanic and mountainous island of Basse-Terre, the soils are ferralsols in the North (AER1), nitisols in the South (AER2), vertisols in the East (AER3) and andosols in the central part (AER4) (Sierra et al. 2015). In the flat island of Grande-Terre, the soils are vertisols (AER5). The wet season runs from August to October and the dry season from January to July. It rains much more in Basse-Terre (mean rainfall of 2300 mm/year) than in Grande-Terre (1100 mm/year). The average daily temperature in Guadeloupe ranges between 23 and 32 °C, with little amplitude between the minimum and maximum temperature during the day (6 °C on average).

### Sampling procedure

The field surveys took place from February to June 2018. The LFS well known on the territory were considered, from family use farmers, traditional farmers to industrial farmers. Information on some LFS in Guadeloupe, in particular livestock in mixed farming systems, is limited, due to the poor administrative organization of these systems (Stark et al. 2016). Consequently, sampling was carried out using a snowball method (Harper et al. 2013). It is a non-probabilistic method where farmers were selected from existing networks between farmers. In this paper, we focus on the three primary species in Guadeloupe that still contain Creole breeds: pigs, beef cattle and goat (Verrier et al., 2015). In a first step, we have contacted farmers using available networks from previous surveys (Gourdine et al. 2010; Gunia et al. 2010; Boval et al. 2012) and from agricultural chambers and cooperatives. In a second step, farmers were found by direct canvassing in the field. The field visits were carried out by AER to ensure that all regions were covered (Sierra et al. 2015). Finally, a total of 106 farmers were investigated. We have undertaken not to disclose the personal data of farmers or animal keepers—who have sometimes unidentified animals and belongs to the informal sector (i.e. the part of the livestock economy that is neither taxed nor monitored by legal structures, Zebus et al. 2004). The information, data and verbatim cited in this paper cannot be linked to a specific individual.

### Data collection

**Fig. 1:**
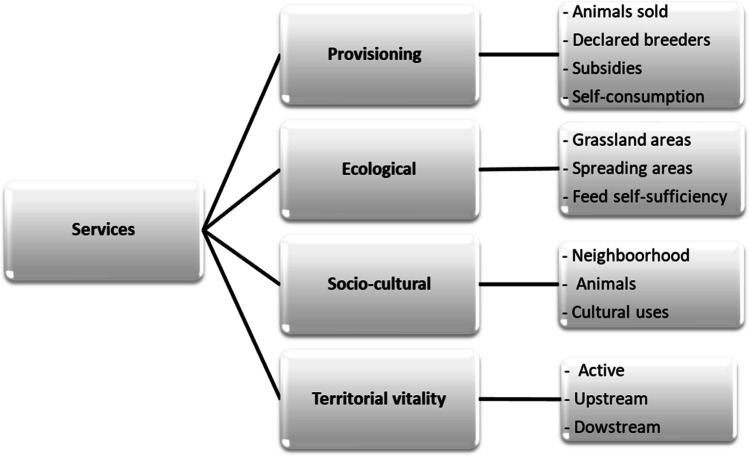
Ecosystem services retained as indicators in the present study (adapted from Ryschawy et al. (2015))

Information was collected using a structured questionnaire as a guideline. The main objective of the interviews was to obtain qualitative and quantitative information for a holistic valuation of the ES provided by LFS. We used the combining biophysical, socio-cultural and economic methods detailed by Ryschawy et al. (2015) to assess the values of the ES. As noted by Ryschawy et al. (2015), there is no a priori prioritization of the ES provided by LFS. The idea was to identify the multiplicity of ES in terms of four bundles of services: provisioning, ecological, socio-cultural and territorial vitality (Fig. 1). The first section was dedicated to the general characteristics of the LFS by collecting socio-economic data (land tenure, labour, household, equipment, sources of income) and crop and livestock activities (land use, crop management, livestock management practices). The information collected about livestock management practices was the management of reproduction, feeding management and health monitoring. The second section of the questionnaire was related to additional questions for a better identification of the four packages of ES (Fig. 1). For provisioning services, additional information was obtained on livestock (number, age, breed and species), the selling price and the type of customers (neighbours, butchers, cooperatives, …). The reasons why farmers choose species and/or breeds were also asked. For territorial vitality, questions were asked about the type of workers directly (active) or indirectly (upstream and downstream from the feed providers to the customers) involved in the livestock activities. For ecological ES, additional information was obtained on the availability of grassland for ruminants and the level of feed self-sufficiency for all species. For socio-cultural services, additional questions were asked about farmers’ perceptions of the neighbourhood’s discomfort caused by the livestock activities and on livestock or livestock products used for cultural ES. The questionnaire was pre-tested with INRAE technical staff who are also farmers.

Surveys were carried out by meeting farmers directly on their farms. The face-to-face interviews were semi-directive: the farmers could follow the questionnaire or they could talk freely. However, some information was crucial for the analysis of ES and the interviewers sometimes needed to redirect the discussion towards this topic. In parallel to the discussion, farmers were recorded on a dictaphone, when they gave their agreement, for keeping the quality and the richness of the interview. After analysing the recordings, if a piece of information was missing or incomplete, the farmer was contacted again by phone or rendezvous, in order to fill in the missing information.

The data and information collected were translated into 12 quantitative indicators of ES following the classification framework from Ryschawy et al. (2015). The panel of indicators was comprised of 4 indicators for provisioning ES, 2 indicators for ecological ES, 3 indicators for socio-cultural ES and 3 indicators for ES related to territorial vitality (Fig. 1). For each studied species, the indicators for provisioning ES were (1) the percentage of animals sold per year relative to the herd size, (2) farm registration (whether a farm was registered in agricultural chamber (= 1) or not (= 0)), (3) the subsidies (whether farmer received subsidies for the studied livestock species production (= 1) or not (= 0)) and (4) the percentage of animals produced for self-consumption relatively to the herd size. The 2 indicators for ecological ES were (5) the percentage of the area dedicated for grasslands relative to the total land surface and (6) the percentage of feed self-sufficiency relative to the feed availability. Feed self-sufficiency represents the ability of farmers to provide feed for livestock farming from their own forage and crop production, which reduces the pressure on the global environment (Pendrill et al. 2019). The 3 indicators used for socio-cultural ES were (7) a subjective rating of appreciation of the farm by the neighbourhood, expressed in percentage, (8) the percentage of live animals involved in cultural process relative to the herd size and (9) the percentage of animals whose products (meat, skin, etc.) were involved in cultural uses. The 3 indicators used for territorial vitality ES were (10) the number of workers on the farm (active), (11) the estimated number of people involved upstream and (12) downstream the livestock production.

### Statistical analysis

All statistical analyses were carried out in the software R (version 3.6.0, R Development Core Team 2019). The effect of breed was analysed by grouping animals into three categories: Creole breed, crossbred animals and exotic breeds. Animals resulting from a cross between a Creole breed and an exotic breed are considered crossbred animals. The data from the surveys were firstly analysed with descriptive statistical analysis. Therefore, a principal component analysis (PCA) was conducted and it was followed by a hierarchical ascending classification (HAC) on the outcomes of the PCAs in order to highlight different groups within species. As input to the PCAs, we used the 12 indicators defined to assess ES (Fig. 1) and the three following variables as illustrative variables: the number of Creole, crossbred and exotic animals. The classification was made for the three species independently of each other. The HAC was made using the Euclidean distance and Ward’s minimum-variance method, which minimizes the decrease of inertia between groups when two groups are gathered into one. The number of farm types within species (i.e. clusters) was defined using the dendrogram shape. We chose to cut the tree at the optimal cut level proposed by the HCPC function of FactoMineR (Le et al. 2008). Each cluster from the three typologies is then analysed individually to draw out indicators of ES with a rose diagram. As proposed by Ryschawy et al. (2015), the 12 indicators of ES were normalized within each cluster. This highly visual representation allows for a quick understanding of the functions ensured by LFS. To evaluate whether Creole breeds provide specific ES, farms composed of Creole animals (more than 80% of the animals present in the farm) were compared to other farms using a multivariate analysis of variance (MANOVA) with the package MANOVA-RM (Friedrich et al. 2018). This procedure was chosen because it provides accurate *p*-values even with small sample sizes using resampling techniques for approximating the sampling distribution (we used 1000 iterations for each model).

## Results

### Characteristics of the livestock farms

**Fig. 2:**
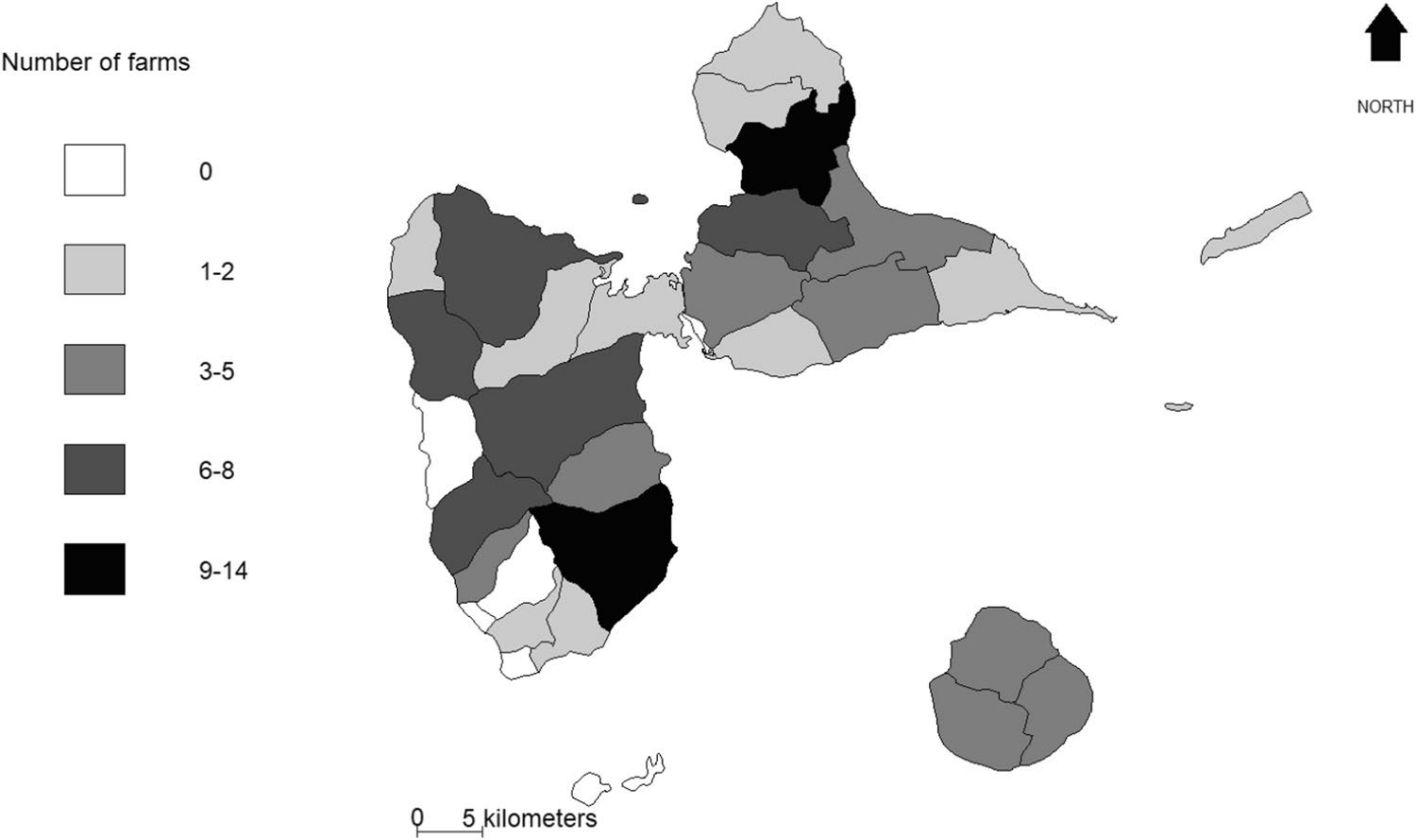
Map of the study area: number of farms that accepted to answer the survey

As illustrated by Fig. 2, the 106 farms covered a large part of the Guadeloupe archipelago and they were from the five agroecological areas of the territory. Farmers were either in livestock intensive system or in the extensive system including animals used for reproduction or only owners of live animals for fattening. Table 1 lists the main characteristics of the 106 farms. Farms that raised pigs, cattle or goats were represented respectively 37.7%, 53.8% and 62.3% of the sample. Monospecific LFS (i.e. LFS consisting of only one livestock species) represent 53.8% of the sample (24.5% with goats, 20.8% with cattle and 8.5% with pigs). Mixed LFS (i.e. LFS consisting of different livestock species) account for 46.2% of the sample. Only 7.5% of the mixed LFS raised the three species studied in this article (pigs, cattle and goats). Mixed LFS that raised only cattle and goats were more encountered in our sample (17% of the sample which corresponded to 36.7% of the mixed LFS sample) than those with cattle and pigs (8.5% of the sample) or goat and pigs (13.2% of the sample).

**Table 1:** Main characteristics of the farmers and their livestock activities

Farmers	Item	Number	Percentage
Gender (*n* = 106)	Male	94	88.7%
	Female	12	11.3%
Age (years; *n* = 106)	20–29	7	6.6%
	30–39	10	9.4%
	40–49	17	16.0%
	50–59	29	27.4%
	Above 60	43	40.6%
Secondary occupation (*n* = 106)	No	19	17.9%
	Employee	53	50.0%
	Unemployed	6	5.7%
	Student	2	1.9%
	Retired	26	24.5%
**Livestock**	**Item**		**Percentage**
Farms (*n* = 106)	With pigs	40	37.7%
	With cattle	57	53.8%
	With goat	66	62.3%
Monospecific farms (*n* = 57)	Pig	9	8.5%
	Cattle	22	20.8%
	Goat	26	24.5%
Mixed livestock farms (*n* = 49)	Pig + cattle	9	8.5%
	Pig + goat	14	13.2%
	Cattle + goat	18	17.0%
	Pig + cattle + goat	8	7.5%
Pig (*n* = 40)	0 animal	66	62.3%
	1–20 animals	37	34.9%
	More 100 animals	3	2.8%
Pig feed self-sufficiency level (% of on-farm feed) (*n* = 40)	Total feed self-sufficiency	6	15.0%
	More than 50%	17	42.5%
	10 to 50%	12	30.0%
	≤ 10%	5	12.5%
Cattle (*n* = 57)	0 animal	49	46.3%
	1–5 animals	17	16.0%
	6–15 animals	16	15.1%
	16–30 animals	14	13.2%
	More than 30 animals	10	9.4%
Pasture for cattle (*n* = 57)	No pasture	5	8.8%
	≤ 1 ha	7	12.3%
	1 to 5 ha	24	42.1%
	5 to 10 ha	12	21.0%
	More than 10 ha	9	15.8%
Goat (*n* = 66)	0 animal	40	37.7%
	1–5 animals	11	10.4%
	6–15 animals	35	33.0%
	16–30 animals	9	8.5%
	More than 30 animals	11	10.4%
Pasture for goat (*n* = 66)	No pasture	6	9.1%
	≤ 1 ha	29	43.9%
	1 to 5 ha	21	31.8%
	More than 5 ha	10	15.2%

**Fig. 3:**
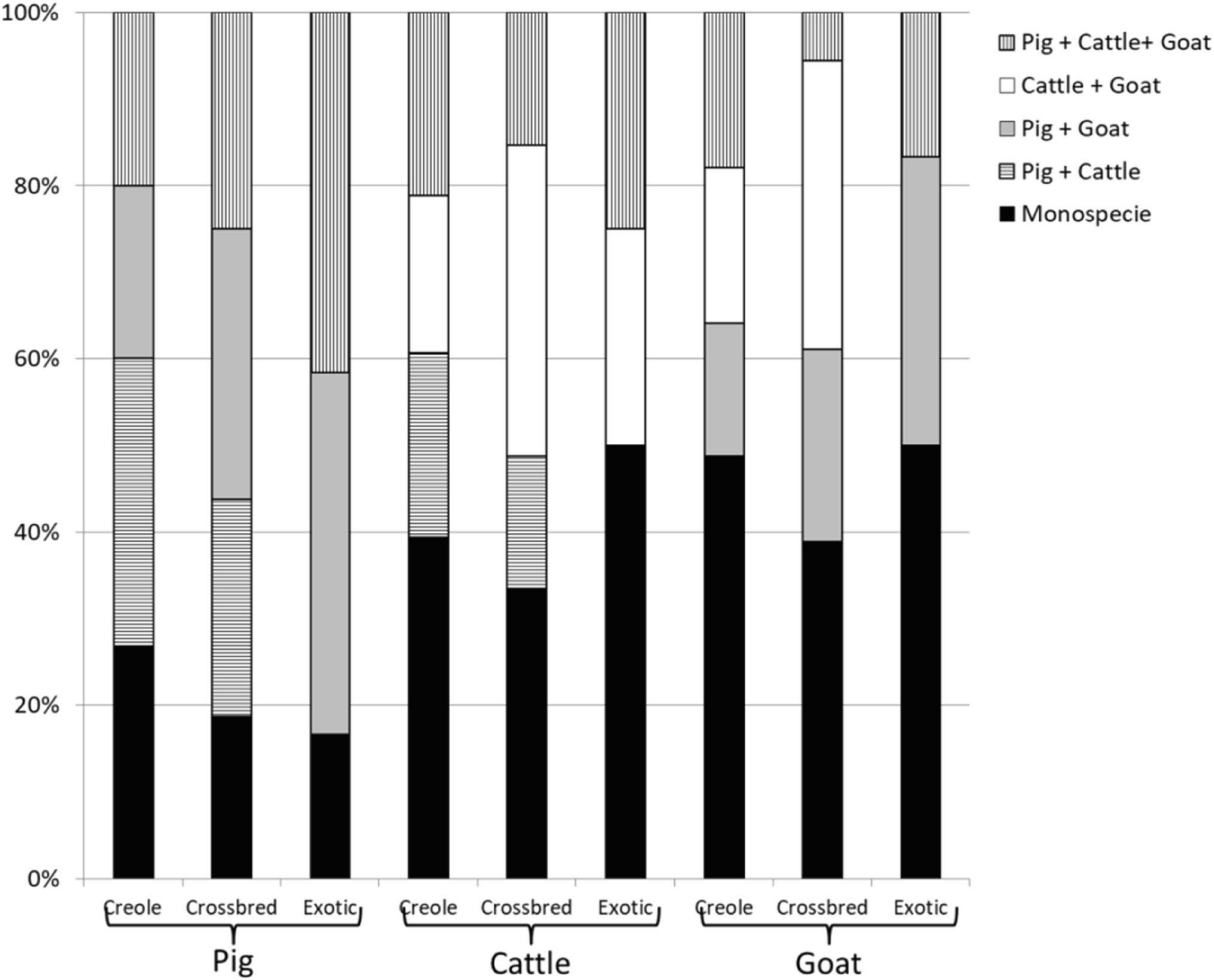
Percentage of farms according to the monospecific (i.e. with only one livestock species) or mixed (i.e. with different livestock species) livestock systems and the genotypes (Creole, crossbred or exotic)

Figure 3 shows the distribution of animals according to LFS (monospecific or mixed) and breed. In our sample, the total number of Creole pigs is lower than the total number of Creole goats or Creole cattle (88 pigs vs. 461 and 486 goats and cattle, respectively). There were 15 pig farms with Creole breed, out of 40 farms with pigs in total. Inversely, the majority of farms in our sample had Creole goats or Creole cattle (58% of the farms).

The following sections report results from the PCAs within species.

### Pig production systems

**Fig. 4:**
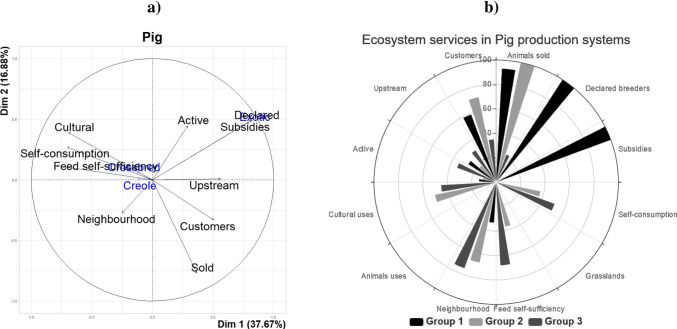
Pig farming systems of Guadeloupe from surveys on a total of 40 farms: **a** first two principal component axis; **b** rose diagram of ecosystem services provided according to the 3 groups of pig farms identified by a principal component analysis followed by a hierarchical ascending classification

The first two principal components (PC) explained 59% of the variability of the pig sub-database (Fig. 4a). The PC1 was determined by the variables “self-consumption”, “feed self-sufficiency” and “cultural uses”, “subsidies” and “declared farmers” (|*r*|> 0.65, *p* < 0.001), while the variables “customers”, “subsidies” and “declared farmers” appeared more correlated to PC2 (|*r*|> 0.63; *p* < 0.01). The illustrative variable “number of exotic pigs” is correlated with these two PC (*r* =  − 0.65 and *r* = 0.67, respectively; *p* < 0.001). The PC3 and PC4 are more explained by the variable “neighbourhood” (*r* = 0.65 and *r* = 0.50, respectively; *p* < 0.001). From the HCA, pig farms have been classified into 3 clusters.

- Cluster 1: The first group was comprised of three intensive pig farming systems (between 631 and 835 pigs from synthetic line Duroc × Pietrain × large white × landrace or purebred large white). Farmers in this cluster aimed at producing 26 weaned piglets per sow per year. The indicators of ES provided by cluster 1 are summarized in Fig. 4b. The provisioning ES (animals sold) was strong, while the values of feed self-sufficiency level and neighbourhood acceptance were low.
- Cluster 2: The second cluster was comprised of 23 farmers. The number of pigs was between 1 and 19 exotic, crossbred or Creole pigs. The main objectives of these farmers were (i) to produce pork for Christmas and other events during the year, (ii) to sell meat and/or animals to friends and to the neighbourhood and (iii) to produce meat for their own consumption. The ES indicators described for cluster 2 (Fig. 4b) are therefore highly developed in animals sold. In this group, much more animals (alive or slaughtered) were sold by the year than the number of pigs present in the farming system.
- Cluster 3: In the third group (14 farmers), 79% of the farmers mainly reared Creole and/or crossbred pigs. Farmers were mainly oriented toward producing pork for their own consumption, and mainly for family events, such as Christmas. Only 14% of these farmers sold pork (1 to 2 pigs per year). This group is characterized by small-scale undeclared pig farms with no public subsidies. Most of the farmers (79%) reared other species (cattle and/or goats, between 3 and 55 animals). The pig housing system was close to the family house (generally in the backyard), either in small pens or tethered to a tree (generally a mango tree). Pigs were usually fed with leftovers, sugarcane, fruits (e.g. breadfruit, mango), food crop residues and concentrates as a complement (between 50 and 100% of on-farm pig feed). The ES indicators described for cluster 4 (Fig. 4b) are therefore highly developed in self-consumption and feed independency.

### Cattle production systems

**Fig. 5:**
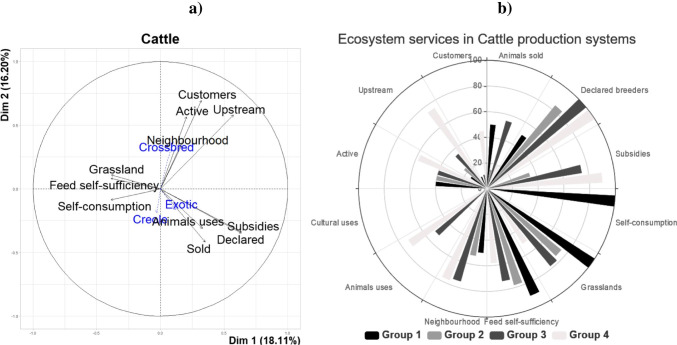
Cattle farming systems of Guadeloupe from surveys on a total of 57 farms: **a** First two principal component axis; **b** rose diagram of ecosystem services provided according to the 4 groups of cattle farms identified by a principal component analysis followed by a hierarchical ascending classification

The first two PC of the PCA for cattle explained 59% of the variability in the cattle sub-database. The variables most correlated with the first two PC were the variables “subsidies” (*r* = 0.64, *p* < 0.001), “declared farmers” (*r* = 0.64, *p* < 0.001) and “upstream” (*r* = 0.57, *p* < 0.001) for PC1, and the variables “customers” (*r* = 0.69, *p* < 0.001), “upstream” (*r* = 0.58, *p* < 0.001) and “actives” (*r* = 0.57, *p* < 0.001) for the PC2. The PC3 is more correlated with the variable “animals sold” (*r* = 0.62; *p* < 0.001) and the PC4 with the variable “self-consumption” (*r* = 0.76; *p* < 0.001). The following HCA resulted in 4 main clusters (Fig. 5a).

- Cluster 1: The first group was comprised of only two farms. These two farms were characterized by a level of self-consumption significantly greater than the level of the other groups (100 vs. 2%; *p* < 0.001). These two farmers reared on average one Creole cattle per year. The ES indicators studied from this group, shown in Fig. 5b in the rose diagram, were developed into ecological issues (grassland uses, feed self-sufficiency). These farmers consumed their own cattle after slaughtering them at the slaughterhouse. Their impact on the downstream sector was low, with few customers as outlets.
- Cluster 2: This cluster gathered 32 farms mainly oriented towards producing Creole cattle (62% of the farms, with 2 to 83 animals). These farmers were mostly in the formal sector (i.e. farmers registered in the agricultural administration for their livestock activities). The majority of farmers in cluster 2 (59%) were also crop producers, mainly in sugarcane or banana production (from 1 to 16 ha). These crop products (sugarcane, banana fruits) and the resulted by-products (leaves, banana pseudostems and nonmarketable fruits) were used as animal feed. As shown in Fig. 5b, the ecological ES related to feeding efficiency was high. In cluster 2, 78% of the feed for cattle came from the farm. However, the provisioning ES was low, related to the low cattle productivity and the small number of animals sold compared to the total number of animals reared. The socio-cultural ES was low related to low neighbourhood acceptance.
- Cluster 3: The third cluster was comprised of 12 farmers. These farms reared mainly crossbred cattle, ranging from 4 to 32 animals. All these farms were administratively declared. The grasslands ranged from 2 to 17 ha. Cattle were mainly tethered, and much of their feed came from the farm (73%). The ES provided by these LFS were summarized in Fig. 4b. Compared to farms in clusters 1 and 2, cluster 3 was characterized by a higher value in socio-cultural ES and a higher value in provisioning ES with more animals sold than in the other groups (55 vs. 28%; *p* < 0.001).
- Cluster 4: The last group was comprised of 11 farmers. These farmers were in the formal sector with large numbers of Creole and crossbred cattle (on average 23 animals per herd). The ES assessment of cluster 4 showed that the ES associated with territory vitality were more important in this group than in other groups, with a level of upstream stakeholders and customers significantly greater than in the other groups (75 vs. 24% and 45 vs. 9%; *p* < 0.001). On the other hand, the values of ecological ES provided by the farms of cluster 4 were lower than in the other cattle farms, related to a lower level of cattle feed self-sufficiency (58 vs. 80%; *p* < 0.05) due to a lower level of grassland used for feeding (51 vs. 84%; *p* < 0.001). In addition, like in cluster 3, farmers of cluster 4 delivered higher socio-cultural ES than farmers from clusters 1 and 2, with animals used for cattle racing and draught oxen.

### Goat production systems

**Fig. 6:**
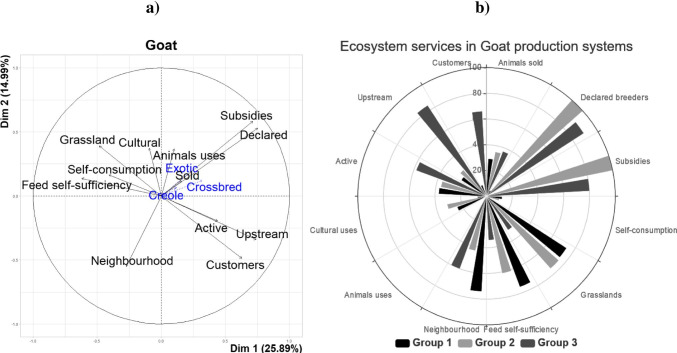
Goat farming systems of Guadeloupe from surveys on a total of 66 farms: **a** first two principal component axis; **b** rose diagram of ecosystem services provided according to the 6 groups of goat farms identified by a principal component analysis followed by a hierarchical ascending classification

The first four PC accounted for 61% of the total variance. The first PC explained 26% of the total variation (Fig. 6a). The variables that contributed most to the first PC were in descending order of importance (*p* < 0.001): “declared farmers” (*r* = 0.75), “upstream” (*r* = 0.74), “subsidies” (*r* = 0.71) and “feed self-sufficiency” (*r* =  − 0.62). The second PC explained 15% of the total variance and was more correlated (*p* < 0.001) with the variables “subsidies” (*r* = 0.58), “declared farmers” (*r* = 0.53) and “neighbourhood” (*r* =  − 0.55). The variables “cultural uses” (*r* =  − 0.63) and “goat sold” (*r* = 0.53) contributed most to PC3 (*p* < 0.001) and the variables “goat sold” (*r* = 0.71) and “active” (*r* =  − 0.58) contributed most to PC4. The HCA discriminated goat farmers into 3 different groups (Fig. 6a).

- Cluster 1: The first group was comprised of 41 farmers who mainly reared Creole and crossbred goats (only 5% of farmers of cluster 1 reared exotic goats) in grassland areas of 2 ha on average. Goat self-consumption was more present in this group than in the other groups (12 vs. 6.5%; *p* < 0.001). Only 7% of these LFS units were administratively declared for goat production. The majority of farmers in this group consumed the meat of their own goats (63% of farmers) and 46% of them slaughtered on the farm for their own personal consumption. Consequently, the ES provided by this group (Fig. 6b) were characterized by a higher socio-cultural ES than in the other groups, with more self-consumption and a great acceptance of the farms by the neighbours. The ecological dimension was also well developed. On the other hand, the value of ES related to territorial vitality was low due to the small number of stakeholders, upstream and downstream in their goat production.
- Cluster 2: The second cluster gathered 15 farms, all of which were officially declared as goat farms in public administration. They all received specific funds from the European Union for rearing small ruminants. These farmers owned on average 30 goats, which was a lucrative activity. The farmers reared mainly Creole or crossbred goats (86%) and a few number of farms reared exotic breeds (21%). The production model was mainly intensive, and the farmers used grasslands of about 7.2 ha on average. Figure 6b describes the ES indicators provided by this second group. Most of the neighbours were unhappy to have this type of farming near to their houses (57 vs. 33% for the other groups; *p* < 0.001).
- Cluster 3: The last goat group is composed of 10 goat farmers mainly with Creole and crossbred goats (only one farmer reared exotic goats). The stocking rate was higher than in the other groups (46.9 vs. 16.2 goats/farm, *p* < 0.001). As illustrated in Fig. 6b, the farms of cluster 3 were poorly involved in socio-cultural ES (6 vs 28% for the other groups; *p* < 0.001). On the other hand, provisioning ES were well developed with a higher proportion of upstream and downstream stakeholders. As illustrated in Fig. 6b, the ES from these farms showed that group 3 was well involved in the goat industry, but their cultural value was reduced, even if the neighbourhood accepted their presence. However, these farms did not have a very positive impact on the environment as the use of grasslands and goat feed independency is limited.

### Breeds and ecosystem services

To analyse the effect of breed on ES provided by LFS, livestock farms with more than 80% Creole animals were compared to farms with more than 80% exotic animals and farms with more than 80% crossbred animals, using MANOVA models (Table 2).

**Table 2:** Effect of breed on ecosystem services using MANOVA models

Specie	N. Creole farms	N. exotic farms	N. crossbred farms	WTS^1^ *p*-value	MATS^2^ *p*-value
Pig^3^	13	11	15	0.18	0.11
Cattle^4^	21	1	27	0.11	0.02*
Goat^5^	29	3	28	0.77	0.54

The response vector for the MANOVA models is composed of the following variables: animals sold, declared breeders, subsidies, self-consumption, grasslands, feed self-sufficiency, neighbourhood, animal uses, cultural uses, active, upstream and customers; ^1^WTS: resampling Wald-type statistic test

^2^MATS: Modified ANOVA-type statistic test

^3^Pig: the 4 variables: declared breeders, subsidies, grasslands and animal uses were excluded from the response vector because of lack of variability

^4^Cattle: the variable cultural uses was excluded from the response vector because of lack of variability

^5^Goat: the variable animal uses was excluded from the response vector because of lack of variability

In pigs, a total of 39 pig farms were compared (13 Creole pig farms, 11 exotic pig farms and 15 crossbred pig farms). The *p-*values obtained by a parametric bootstrap procedure are greater than 0.10, suggesting that there is no breed effect on the ES indicators obtained within pig farms.

In cattle, only Creole farms were compared with crossbred farms because there was only one farm with a herd containing more than 80% of exotic cattle. The WTS *p*-value is greater than 0.11 but the MATS *p-*value is lower than 0.05, indicating that there are significant differences in the ES indicators obtained by these two groups.

In goats, only Creole farms were compared with crossbred farms because there were only 3 farms with a herd containing more than 80% of exotic goats. With WTS and MATS *p-*value greater than 0.5, the MANOVA indicates that there is no significant breed effect on the different ES provided by farms with Creole or crossbred goats.

## Discussion

Livestock practices directly generate ecosystem services, defined as the benefits that humans derive from ecosystems (Leemans 2009). Methods for identifying and valuing the ecosystem services (ES) provided by livestock were studied by different authors (Ryschawy et al. 2015; Bernués and Martin-Collado 2019; Dumont et al. 2019). In line with the combining methods reviewed by Bernués et Martin-Collado (2019), Ryschawy et al. (2015) proposed a list of quantitative and qualitative indicators to measure the ES provided by livestock that could be easier to implement than the Millennium Ecosystem Assessment recommendations (Reid et al. 2005). This list was used in the present paper to assess ES provided by the diversity of LFS studied.

### Characteristics of the livestock farms

In this paper, we focused only on three species (pig, cattle and goat) reared in LFS in Guadeloupe because Creole breeds are well characterized in these species (Naves et al. 2001; Gunia et al. 2010; Gourdine et al. 2010), and these species were the main driver of meat production in Guadeloupe as they accounted for 76% of the total meat produced (Agreste, 2019). Our study confirmed the broad diversity of livestock farming systems in Guadeloupe (Fanchone et al 2020), including the range of livestock activities, the crop-livestock integration and the livestock diversity. In our analysis, farmers with a low number of animals were considered because small LFS (such as family farms) represent the traditional practice in the Caribbean since the time of slavery and these practices remain in Guadeloupe, particularly in semi-urban and rural areas (Xandé 1999; Sainton et al. 2012). Therefore, these LFS cannot be ignored due to their particular functions for household sustainability, as it was shown in some tropical regions (Mbuthia et al. 2015; Gourdine et al. 2018; Huyen et al. 2019).

Our results for both monospecific (i.e. LFS with only one livestock specie) or mixed crop-livestock systems suggest that the intensity of combining livestock species or crop-livestock is low in the majority of LFS studied. As observed by Stark et al. (2016), mixed systems are more time-consuming than monospecific ones, and these multipurpose systems are encountered more in small-scale LFS than in large business-oriented LFS. However, the coexistence of this diversity of LFS in Guadeloupe may provide multiple ES.

### The ecosystem services supplied by livestock farming systems in Guadeloupe

The following sections discuss the ES provided by LFS within species.

#### Pig farming systems

Our study showed that the ES provided by pig LFS differed significantly between the 3 clusters identified, which could be attributed to the different purposes of farmers in rearing pigs. Farmers in the first cluster followed the intensive production rules of the European Union (feeding with industrial concentrates, subsidies for production help, building, batch management, etc.). The provisioning ES was consequently strong while the values of feed self-sufficiency level, which summarizes the pressure on the environment and the neighbourhood acceptance, were low. Similarly, Ryschawy et al. (2017) reported greater levels of provisioning services in high pig density farms and lower ecological services. In the present study, LFS of the last two clusters provide all types of ES studied. These LFS were small-scale pig farming systems characterized by a limited investments for pig breeding. In our study, cultural activity was considered the primary cultural ES in these small-scale pig farms. In addition, pigs are also present to ensure an income by the sale of meat or piglets. The pig farming practices were part of the cultural use of ham and pork meat sales to the neighbourhood during the Christmas period. Indeed, the consumption of pork meat in Guadeloupe is very important during Christmas (stew, smoked ham with sugar cane, …) and consumers distinguish between pork produced by small-scale farms in more environmentally friendly circumstances and the one coming from high pig density farms. Furthermore, with the integration of customers in the neighbourhood, these small-scale pig farmers impacted the local circular economy. In direct sale systems, after the carcass has been cut up, the meat is sold in 2.5 kg batches, with a mixture of parts of the carcass (Zebus et al. 2005). The meat mass is often accompanied by a special recipe for black pudding (made with pig blood). These two small-scale pig farming groups showed main differences in the provisioning services. These ES were greater in the second cluster. The differences could be attributed to the fact that the LFS of cluster 2 were small-scale growing-finishing pig production systems as farmers bought a few young pigs from other farmers and reared them to final live weight before slaughter. Finally, in our study, it was difficult to assess the ecological ES provided by pig farming related to the use of pig manure for fertilization. In the present study, the practice of pig manure recycling is rarely used in farmers’ practices. Similar conclusions were found in Mbuthia et al. (2015) when they studied pig farming systems in Kenya and in Fanchone et al. (2020) when they compared the diversity of agroecological practices in Martinique, whereas pig manure recycling was found to be frequently used in Vietnam (Huyen et al. 2019).

#### Cattle farming systems

In our study, cattle farms had common ecological ES related to management in grass-based systems either in grasslands or in small-scale rotational grazing. In Guadeloupe, rearing tethered animals and using rotational management are very frequent in cattle farmers. Boval et al. (2012) showed how cattle tethering is an original tool for natural resources management in Guadeloupe as they maintain meadows and roadsides and they utilize undivided lands and land unsuitable for cultivation. Ryschawy et al. (2015) observed that ruminant farms provide more ecological services than farms with other species because cattle grazing allows the conservation of floral and faunal diversity that would close over in the absence of cattle. Our results obtained through the farm-level survey are in accordance with Ryschawy et al.’s (2015) study, as grasslands are important components of the Guadeloupe landscape.

The levels of ES provided by cattle LFS varied among the 4 clusters identified, mainly in ES related to socio-cultural services and territorial vitality. The socio-cultural services provided by cattle are mostly cart drawn by oxen and draught oxen, mainly from two clusters of farmers. The socio-cultural ES was low in the other clusters either because the cultural acceptance of the farms was moderate, as the neighbourhood complained or because the few cattle reared were for self-consumption. In the present study, cattle were the only species for which live animals were used in the cultural service (cart race). When investigating the ES provided by livestock in some European regions, Ryschawy et al. (2015) showed that cattle provided more ES linked to cultural services than other species. However, in the present study, the cultural value associated with pig and goat farms was greater than those linked to cattle. This discrepancy could be explained by the richness of European cattle quality labels.

As observed in this study, the highest values in provisioning services were associated with the largest farms. This is consistent with previous studies (Dumont et al. 2019), showing the importance of large cattle farms in direct or indirect employment in the agricultural sector.

In the present study, cluster 1 was composed of only two farmers that were very different from the others, as the main ES provided by their cattle production was provisioning services for self-consumption. The current literature on cattle family farms in Guadeloupe orientated to beef self-consumption is scarce, but it is part of the reality of cattle production in Guadeloupe.

#### Goat farming systems

In our study, goats were found in at least three different rearing systems: intensive, extensive and in small numbers tethered to stake or in pens. They were present throughout Guadeloupe. Compared with the ES provided by a pig of cattle production, goat farms provided a higher diversity of ES, irrespective of systems. The first cluster was more heterogeneous than the others because breeders had several different objectives. For the smaller farmers of the first cluster, goat farming was mainly a “hobby” in addition to their financial objectives. For larger farmers, goat farming was seen as essential, a way to earn money. It should be noted that for some of the small farmers in this group who were unemployed (34%), rearing goats also took a fundamental dimension as a means of survival, as “no other income goes into the household”. In the second cluster, goats were found to be culturally important. The animals were often slaughtered for Catholic holidays (Easter, Christmas and Pentecost) or sold or sacrificed for Hindu ceremonies. Alexandre et al. (2003) showed that goats are associated with religious festivals and these practices impact the price of goat meat either in the formal or in the informal sector. Furthermore, goat skins are an important element of the traditional musical drum used to play Gwoka, a drum-based music and dance inscribed on the UNESCO’s list of the representative intangible cultural heritage of Humanities (Camal 2016). For the majority of this group, regardless of the size of the farm, goats were above all, a source of “pleasure” that was often rooted in a family tradition. Consequently, the socio-cultural services provided by goats could be considered an important part of the African and Indian cultural heritage of Guadeloupe. On the opposite, the socio-cultural ES of the second cluster were lower than in the other groups, mainly because most of the neighbours were unhappy to have this type of farming near to their houses. The goat farmers in the second and third clusters were strongly involved in the goat industry of Guadeloupe. Consequently, the services provided by these clusters were more oriented towards provisioning services, with high proportions of upstream and downstream stakeholders, than in cultural values. However, these farms did not have a very positive impact on the environment as the use of grasslands and goat feed independency were limited. As suggested by Dumont et al. (2019), high levels of ES cannot be obtained simultaneously for all service bundles because of possible antagonism between them, for instance, between provisioning and ecological services.

### Breed and ecosystem services: is there breed specificity?

In contrast to our expectations, Creole pig and Creole goat breeds did not provide specific ES compared to exotic and crossbred livestock. This result confirms the low correlations found between the illustrative variables (Creole, exotic and crossbred genotypes) and the principal components in the PCA analysis. These results could be explained by the lack of additional or differential value given to Creole breeds. In general, ES provided by specific breeds are connected to labelled products allowing the promotion of specific characteristics of the breed in the marketing system (Bernués and Martin-Collado 2019). Up to now, there are no specifications (e.g. protected designation of origin) in Guadeloupe to differentiate management between breeds. In the present study, the method used to assess ES provided by livestock did not allow emphasis on distinct characteristics of Creole pigs meat qualities (Xandé et al. 2009) and in heat tolerance (Renaudeau et al. 2006), nor of Creole goats meat qualities (Limea et al. 2012) and gastrointestinal parasitic nematodes resistance (Mandonnet et al. 2006). Consequently, the ES identified mainly depend on the goal of pig or goat farmers and the way they managed their livestock production. Contrary to pig and goats LFS, we found that Creole cattle provided significantly different ES (for one of the 2 statistical tests) than crossbred cattle. The differences were explained by higher self-consumption and uses of cattle in cultural process and lower upstream and customers in Creole cattle farms than in crossbred cattle farms. This is probably due to the traditional use of Creole cattle for draught and in traditional cattle cart race competition. Furthermore, historically, Creole cattle have been selected for their draught characteristics (Gautier and Naves 2011). The cart race activities are increasingly carried out with crossbred or exotic animals with higher muscle development than Creole cattle. However, Creole cattle are still used more for a draught of sugar cane carts or ploughing because of their better endurance in long-term effort than crossbreed or exotic breeds (Versini 1997).

## Conclusion

The present study demonstrates that the ecosystem services provided by livestock farming systems in the tropical conditions of Guadeloupe are multiple. Our study, by assessing and comparing ecosystem services provided by pig, cattle and goat systems, covers the larger part of the meat sectors in Guadeloupe. We found differences in services provided between species. We highlight that ES indicators are not breed dependent but rather livestock farming system dependent because ES provided by livestock rely more on the rearing management than on the breed type. Further studies involving assessment of ES at the territorial level combining farmers and other stakeholders should provide information to improve ES provided by livestock in Guadeloupe. Such studies could facilitate collective initiatives involving political decision-makers to develop a specific niche market that promotes the recognized qualities of Creole breeds, and help their conservation.

## Data Availability

The datasets generated and analysed during the current study are available from the corresponding author on reasonable request.
